# Improving influenza prediction in Quanzhou, China: an ARIMAX model integrated with meteorological drivers

**DOI:** 10.3389/fpubh.2025.1662775

**Published:** 2025-10-22

**Authors:** Huanhuan Pan, Jiangyi Liu, Fengping Li, Weiming Wang, Xinlan Huang, Huanrong Li, Xiaoxiong Yang, Xueqi Chen

**Affiliations:** ^1^The Affiliated Quanzhou Center for Disease Control and Prevention of Fujian Medical University, Quanzhou, China; ^2^Quanzhou Center for Disease Control and Prevention (Quanzhou Health Supervision Institute), Quanzhou, China; ^3^The School of Public Health and Medical Technology, Xiamen Medical College, Xiamen, China

**Keywords:** influenza-like illness, DLNM modeling, ARIMAX modeling, meteorological determinants, prediction

## Abstract

**Background:**

Influenza remains a significant public health challenge, characterized by substantial seasonal variation and considerable socioeconomic burden. Although meteorological factors are known to influence influenza transmission, their specific effects within subtropical monsoon climates, such as that of Quanzhou, remain inadequately characterized.

**Methods:**

We analyzed weekly influenza-like illness (ILI%) data from sentinel hospitals in Quanzhou between 2016 and 2024. Descriptive statistics, distributed lag nonlinear models (DLNM), cross-correlation function (CCF) analysis, and ARIMAX modeling were employed to examine the lagged and nonlinear associations between meteorological variables and ILI%.

**Results:**

The overall ILI% during the surveillance period was 2.32%, with significant temporal trends: a pronounced decline from 2016 to 2020 (APC = −22.693, *p* = 0.001) was followed by a significant increase from 2020 to 2024 (APC = 21.555, *p* = 0.003). Children under 15 years of age were the most affected demographic. A consistent bimodal seasonal pattern was observed, with a primary peak in winter (weeks 50–14) and a secondary peak in summer (weeks 20–29). DLNM analysis indicated that low atmospheric pressure (<997 hPa) at lag 0–0.5 weeks was associated with increased ILI risk, while higher pressure (≥1,010 hPa) had a protective effect (relative risk [RR] = 0.85, 95% CI: 0.73–0.99). Precipitation of 4–16 mm elevated ILI risk (RR = 1.10, 95% CI: 1.02–1.18), whereas precipitation >21 mm was protective. Wind speed demonstrated an N-shaped association with ILI%, though this was not statistically significant. The optimal forecasting model incorporated precipitation at a 2-week lag as an exogenous variable (ARIMA(1,1,1)(1,1,1)_52_ + WAP(lag2)) and yielded the highest predictive accuracy for 2024 ILI%, improving RMSE by 2.9% and MAE by 1.3% compared to the baseline model.

**Conclusion:**

Incorporating meteorological factors significantly improves the accuracy of influenza forecasting models. These findings support the development of climate-informed early warning systems and targeted public health interventions in subtropical regions.

## 1 Introduction

Influenza is an acute viral respiratory infection that affects populations across all age groups worldwide and causes substantial mortality during pandemics, seasonal epidemics, and sporadic outbreaks. It is estimated that influenza viruses infect approximately 10% of the global population each year, resulting in around 500,000 annual deaths. These figures underscore the considerable public health burden imposed by influenza on a global scale (1). In temperate climates, seasonal epidemics occur mainly during winter, while in tropical regions, influenza may occur throughout the year, causing outbreaks more irregularly (2). Recent epidemiological anomalies—including the intensification of summer influenza activity in subtropical zones and the global disappearance of the B/Yamagata lineage-present fresh challenges for health authorities and influenza surveillance efforts (3). This emerging trend presents fresh challenges for health authorities and influenza surveillance efforts. A growing body of evidence suggests meteorological factors significantly modulate influenza dynamics; however, their effects exhibit striking regional heterogeneity (4–6). For instance, low temperatures and humidity in temperate zones may enhance viral stability, whereas monsoon-driven climate systems-characterized by abrupt humidity shifts and temperature fluctuations in subtropical regions like Quanzhou-could distinctly alter transmission pathways.

Quanzhou, a coastal prefecture-level city located in southeastern Fujian Province, China, features a typical subtropical monsoon climate characterized by mild winters, hot and humid summers, and abundant annual precipitation. The region experiences distinct seasonal variations in wind patterns, humidity, and temperature, heavily influenced by the western Pacific Ocean and the East Asian monsoon system. Despite these pronounced climatic characteristics, systematic investigations into climate-influenza interactions within subtropical monsoon climates remain scarce. This gap is particularly critical in regions like Quanzhou, where humid summers and mild winters may drive unique epidemic behaviors, yet predictive models tailored to such environments are lacking.

This study employs a dual analytical framework to advance influenza forecasting in subtropical monsoon climates. First, we quantify the non-linear and lagged effects of meteorological factors on Influenza-Like Illness Percentage (ILI%) using Distributed Lag Non-linear Models (DLNM). Second, by integrating Cross-Correlation Function (CCF) analysis to identify optimal lag orders, we construct an Autoregressive Integrated Moving Average with Exogenous Variables (ARIMAX) model that synergizes climatic drivers with historical ILI% data. This approach not only captures complex climate-epidemiology interactions but also generates actionable predictions for public health planning.

## 2 Materials and methods

### 2.1 Study area and data sources

Quanzhou, situated in the southeast coastal area of China, covers a total area of 11,295.57 square kilometers. By the end of 2024, the city had a permanent resident population of 8.91 million. The region experiences a subtropical monsoon climate, characterized by humid summers, mild winters, and significant seasonal precipitation variability. Weekly data on Influenza-Like Illness (ILI) counts and ILI% from January 2016 to December 2024 were obtained from the China Disease Prevention and Control Information System.

The meteorological data utilized in this study encompassed eight indicators sourced from the China Meteorological Data Network, comprising weekly average temperature (WAT,°C), maximum temperature (MaxT,°C), minimum temperature (MinT,°C), weekly average barometric pressure (WABP, hPa), weekly average relative humidity (WARH, %), minimum relative humidity (MinRH, %), weekly average precipitation (WAP, mm), and weekly average wind speed (WAWS, m/s). The raw values of these meteorological variables were used directly in all subsequent analyses without normalization or standardization, to preserve the interpretability of results in their original physical units.

### 2.2 Statistical analysis

#### 2.2.1 Seasonal variation characterization

A seasonal index was calculated using Microsoft Excel 2017 to quantify influenza seasonality, defined as (Equation 1):


(1) $$\begin{array}{l} \text{Seasonal Index}=(\begin{array}{l} \text{Average}\;\mathrm{ILI}\%\text{for}\;a\;\\ \text{Specific Week}\\ \left(2016–2024\right) \end{array})/\\ \left(\text{Overall Average Weekly}\;\mathrm{ILI}\%(2016–2024)\right) \end{array}$$

Weeks with indices >1 were classified as high-incidence periods, while values near or below 1 indicated non-significant seasonality (7).

#### 2.2.2 Trend analysis

Temporal trends in ILI% were analyzed using Joinpoint 5.4.0. The annual percentage change (APC) quantified segment-specific trends, while the average annual percentage change (AAPC) assessed overall trends. APC > 0, the incidence of the disease is increasing in that time period; conversely, if APC < 0, the incidence is decreasing in that time period. APC = AAPC indicates a monotonically increasing or monotonically decreasing trend (8).

#### 2.2.3 Data processing and correlation analysis

Descriptive statistics for ILI, ILI%, and meteorological variables were computed using R software (version 4.4.3, R Foundation for Statistical Computing) with the following packages: dplyr for data manipulation and forecast for time-series decomposition. Normality of ILI% and meteorological variables was assessed using the Shapiro–Wilk test (shapiro.test function from the stats package). Both ILI% and meteorological data showed non-normal distributions (all *p* < 0.05), supporting the use of nonparametric Spearman’s rank correlation analysis (implemented via cor.test with method = “spearman”). All statistical tests were two-tailed, and a significance threshold of *p* < 0.05 was applied. Correlations between ILI% and eight meteorological factors were examined. To mitigate multicollinearity, variables with Spearman’s correlation coefficients > 0.7 were excluded from concurrent inclusion in subsequent multivariate models (9).

#### 2.2.4 Distributed lag nonlinear modeling

The influence of meteorological factors on ILI% has a lagged effect (10). The DLNM can take into account the nonlinear exposure-response relationship and the lag effect of exposure factors on outcomes simultaneously (11, 12). It has been widely used to evaluate the expose-lag-effectcorrelations between meteorological factors and infectious diseases, such as dengue, mumps, and acute respiratory infections (13–15).

We modeled the association between meteorological variables and ILI% using a quasi-binomial regression within the DLNM framework, implemented with the dlnm package in R. The model specification was as follows (Equation 2):


(2) $$\text{logit}(p)=\beta_{0}+\mathrm{CB}(X_{4})+\mathrm{CB}(X_{7})+\mathrm{CB}(X_{8})+\mathrm{ns}(\text{Time},\mathrm{df}=63)$$

p represents the proportion of ILI cases among the total outpatient consultations for a given week. $\mathrm{CB}(X_{4})$ , $\mathrm{CB}(X_{7})$ , and $\mathrm{CB}(X_{8})$ denote the cross-basis functions for WABP, WAP, and WAWS, respectively. These functions simultaneously capture the potentially nonlinear exposure-response associations and the delayed effects (lag-response) of each meteorological variable. Each cross-basis was constructed using natural cubic splines with 3 degrees of freedom for both the exposure-response and lag-response dimensions. A maximum lag of 3 weeks was specified *a priori* to capture the short-term delayed effects of weather on ILI transmission. The choice of this lag period is epidemiologically justified, as it accommodates the sequential timeline from environmental exposure (e.g., to weather conditions) to the development of symptoms (1–4 days incubation period), subsequent care-seeking behavior, and final reporting as an ILI case. Furthermore, a three-week window is sufficient to capture secondary transmission cycles within households or communities (16, 17). The term ns(Time,df=63) represents a natural cubic spline of time with 63 degrees of freedom. This term was included to control for unmeasured temporal confounders, including long-term trends, seasonality, and other time-varying factors not explicitly measured in the model. The degrees of freedom were set at 7 per year for the 9-year study period (2016–2024), providing a flexible adjustment for the underlying temporal structure of the data. $\beta_{0}$ is the model intercept. The model was fitted using maximum likelihood estimation. The results are presented as relative risks (RRs), derived by comparing the predicted risk at specific exposure levels against the reference value (the median value of each meteorological variable).

Model parameters, including the degrees of freedom for splines and the lag structures, were optimized based on the Akaike Information Criterion (AIC). Meteorological factors that showed significant correlations (*p* < 0.05) in the preliminary correlation analyses were incorporated into the DLNM to quantify their lag-specific effects on ILI%.

#### 2.2.5 SARIMA and ARIMAX modeling

The Autoregressive Integrated Moving Average (ARIMA) model, first proposed in 1976 (18), is now widely used in the prediction and early warning analysis of infectious diseases. For time series data exhibiting periodic patterns, the Seasonal Autoregressive Integrated Moving Average Model (SARIMA) combines seasonal differencing with the standard ARIMA model, making it well-suited for modeling data with recurring characteristics. The SARIMA model is abbreviated as ARIMA(*p*,*d*,*q*)(*P*,*D*,*Q*)*s*, where s denotes the seasonal period, and *p*(*P*), *d*(*D*), and *q*(*Q*) stand for the unseasonal (seasonal) autoregressive, difference, and moving average orders, respectively. The determination of these parameters, (*p*,*d*,*q*) and (*P*,*D*,*Q*), is achieved through an analysis of the Partial Autocorrelation Function (PACF) and the Autocorrelation Function (ACF). To enhance predictive accuracy, the ARIMAX model extends the SARIMA framework by incorporating exogenous variables—such as meteorological factors—that influence the target series (19).

All analyses were performed using R 4.4.3 with packages forecast (for auto.arima and model fitting), tseries (for stationarity tests), and dlnm (where applicable). All statistical tests were two-tailed, with significance set at *p* < 0.05.

The weekly ILI% data from January 2016 to December 2023 was utilized as the training set for model development, while data from January to December 2024 was reserved as a test set for external validation of model generalizability. The modeling procedure consisted of three main phases:

1. Stationarity Assessment and Differencing: The stationarity of the ILI% time series was rigorously assessed using the Augmented Dickey-Fuller (ADF) test and a Kwiatkowski-Phillips-Schmidt-Shin (KPSS) test. The appropriate non-seasonal differencing order (*d*) was determined through the ndiffs() function in R, which identifies the minimum number of differences required to achieve stationarity. Seasonal differencing order (*D*) was determined by examining the ACF plot for persistent seasonal patterns at lag 52, corresponding to the yearly cycle.
2. The orders of the autoregressive (*p*), moving average (*q*), and their seasonal counterparts (*P*, *Q*) were identified through a complementary approach that combined graphical analysis and automated algorithm selection. Initial guidance for appropriate orders was obtained through visual inspection of the ACF and PACF plots of the differenced series. This graphical analysis was then validated and refined using the auto.arima() function in R, which was employed with comprehensive search settings (stepwise = FALSE, approximation = FALSE) to systematically compare a wide range of candidate models. The final model selection between these candidates was based on the minimization of the AIC and the Bayesian Information Criterion (BIC).
3. Following parameter estimation, model adequacy was thoroughly evaluated through residual diagnostics and error metrics, wherein the Ljung-Box test was applied to assess whether model residuals approximated white noise (i.e., exhibited no significant autocorrelation), while model fit was quantified using the Root Mean Square Error (RMSE) on the training data.

After establishing an optimal univariate SARIMA model, meteorological factors significantly associated with ILI% were identified and incorporated as external regressors into a multivariate ARIMAX framework. The CCF analysis was applied to determine optimal lag orders for these predictors. The final ARIMAX model’s performance was evaluated against the baseline SARIMA model using both goodness-of-fit criteria (AIC/BIC) and forecast accuracy metrics on the test set to demonstrate any improvement in predictive capability.

## 3 Results

### 3.1 A description of ILI and ILI% characteristics

**Figure 1:**
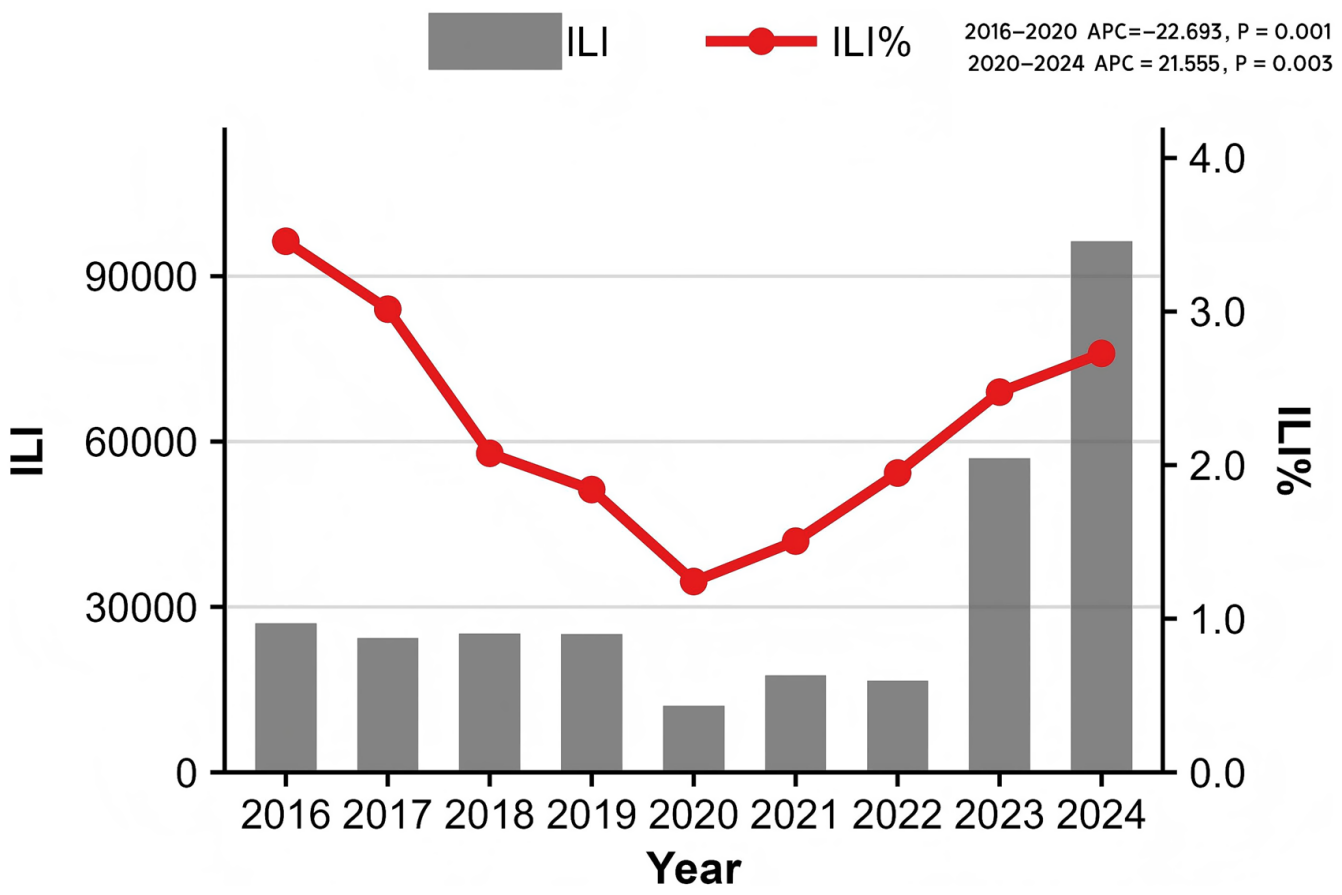
Sentinel site monitoring in Quanzhou, 2016–2024.

From 2016 to 2024, sentinel hospitals in Quanzhou City reported a total of 12,991,585 outpatient and emergency visits, including 301,239 ILI cases, yielding an overall ILI% of 2.32%. Notably, ILI% exhibited marked temporal variability: the highest rate (3.46%) occurred in 2016, while the lowest (1.24%) was recorded in 2020. Joinpoint regression analysis revealed distinct trend phases (Figure 1), which showed a decreasing trend in ILI% from 2016 to 2020 (APC = −22.693, *p* = 0.001) and an increasing trend from 2020 to 2024 (APC = 21.555, *p* = 0.003).

#### 3.1.1 Population distribution

**Figure 2:**
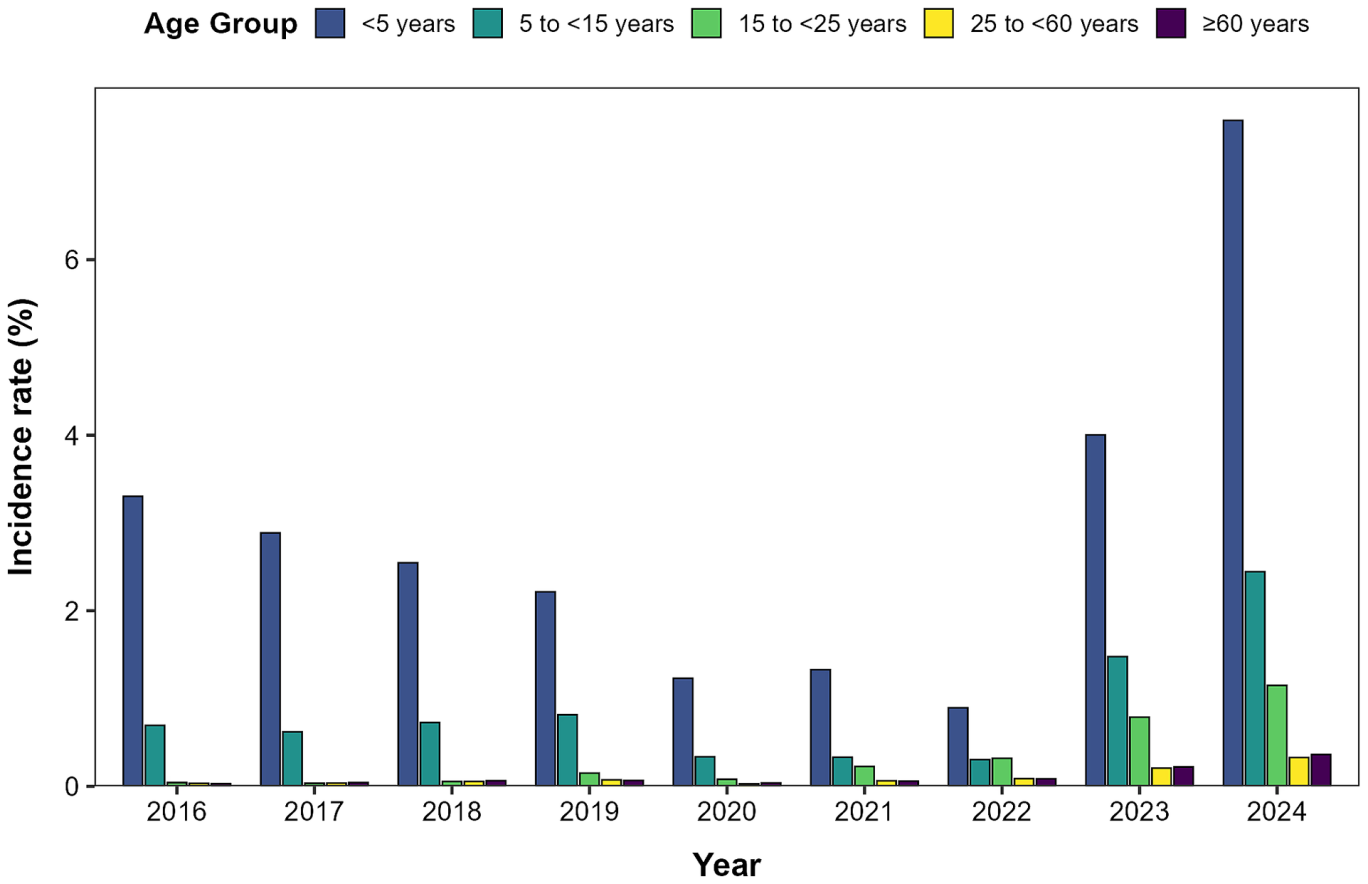
Distribution of influenza-like case incidence by age group in Quanzhou City, 2016–2024.

Surveillance data from 2016 to 2024 revealed distinct age-specific patterns in ILI incidence across Quanzhou City. Children under 5 years of age exhibited the highest ILI incidence rate, followed by those aged 5 to <15 years (Figure 2).

#### 3.1.2 Time distribution

**Figure 3:**
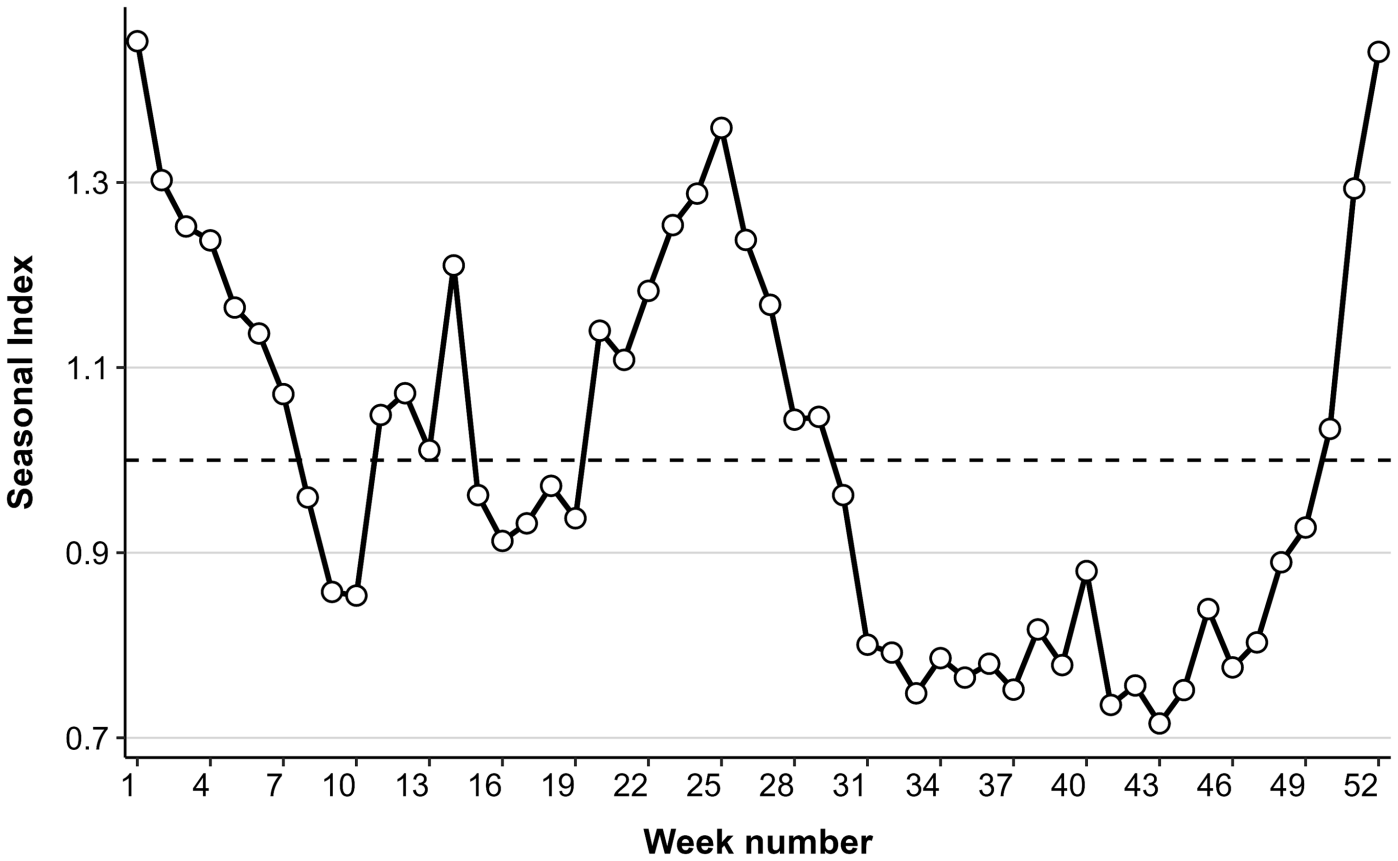
Seasonal index trends.

Analysis of the seasonal index indicated a consistent annual bimodal seasonality in ILI% (Figure 3), featuring two distinct peaks each year. A primary winter peak occurred from week 50 to week 14 (December to February of the following year), which was characterized by markedly higher intensity and prolonged duration. A secondary summer peak was observed between weeks 20 and 29 (May to July), with moderate magnitude in comparison.

### 3.2 Impact of meteorological factors on ILI%

**Figure 4:**
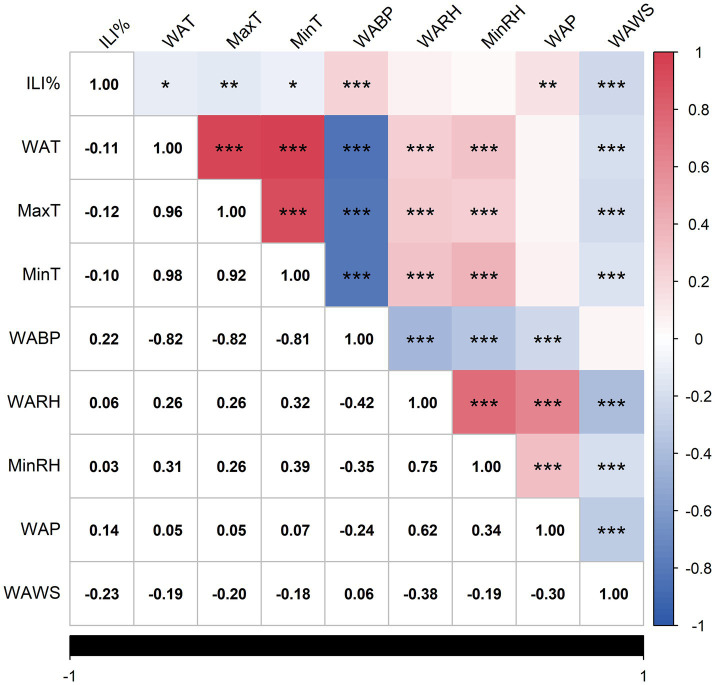
Heat map of correlation analysis. Statistically significant correlations are marked with asterisks (*p < 0.05, **p < 0.01, ***p < 0.001), as indicated in the footnote. Non-significant correlations (*p* ≥ 0.05) are left unmarked.

Spearman’s correlation analysis revealed significant associations between ILI% and multiple meteorological variables. The correlation of the variables in the data was visualized and the heat map obtained is shown in Figure 4, which reveals that ILI% shows a statistically significant correlation with WAT, MaxT, MinT, WABP, WAP, and WAWS.

The negative correlation coefficients between ILI% and WAT(*r* = −0.11), MaxT(*r* = −0.12), MinT(*r* = −0.10), and WAWS(*r* = −0.23) suggest that cooler temperatures and lower wind speeds may elevate influenza transmission risks in Quanzhou. The correlation coefficients between ILI% and WABP, WAP are 0.22 and 0.14 respectively, indicating that the higher the air pressure or precipitation, the higher the likelihood of influenza. The average precipitation (WAP) was 3.24 mm, and the average relative humidity (WARH) was 75.11% (Table 1), providing a quantitative baseline for the local humid subtropical climate that characterizes Quanzhou.

**Table 1:** Descriptive statistics of meteorological factors.

Meteorological Factors	Range	Minimum	Maximum	Average
WAT	23.99	7.07	31.06	21.68
MaxT	28.70	10.50	39.20	28.95
MinT	27.70	0.10	27.80	16.79
WABP	37.39	982.54	1019.93	999.28
WARH	45.83	50.00	95.83	75.11
MinRH	69.00	12.00	81.00	44.39
WAP	38.27	0.00	38.27	3.24
WAWS	8.02	1.47	9.49	4.35

Significant multicollinearity ( ∣r∣ >0.7) was observed among WAT, MaxT, and MinT, as well as between WABP and temperature variables. To address collinearity and prioritize predictive relevance, only meteorological factors exhibiting the strongest associations with ILI% were retained. Consequently, WABP, WAP, and WAWS were selected as exogenous variables for the final model.

### 3.3 Associations between meteorological factors and ILI% identified by DLNM

#### 3.3.1 Effect of WABP on ILI%

**Figure 5:**
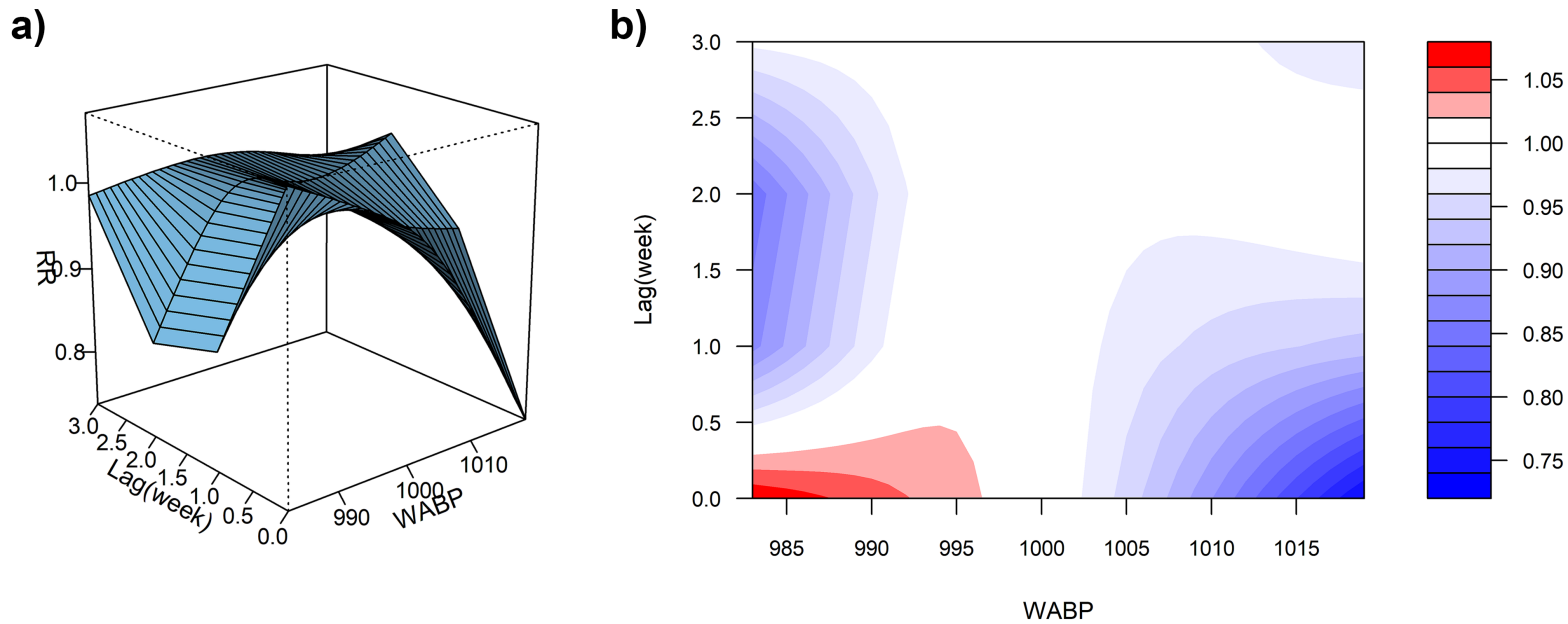
**(a)** 3D graph of WABP-lag-ILI% association; **(b)** contour graph of WABP-lag-ILI% association.

**Figure 6:**
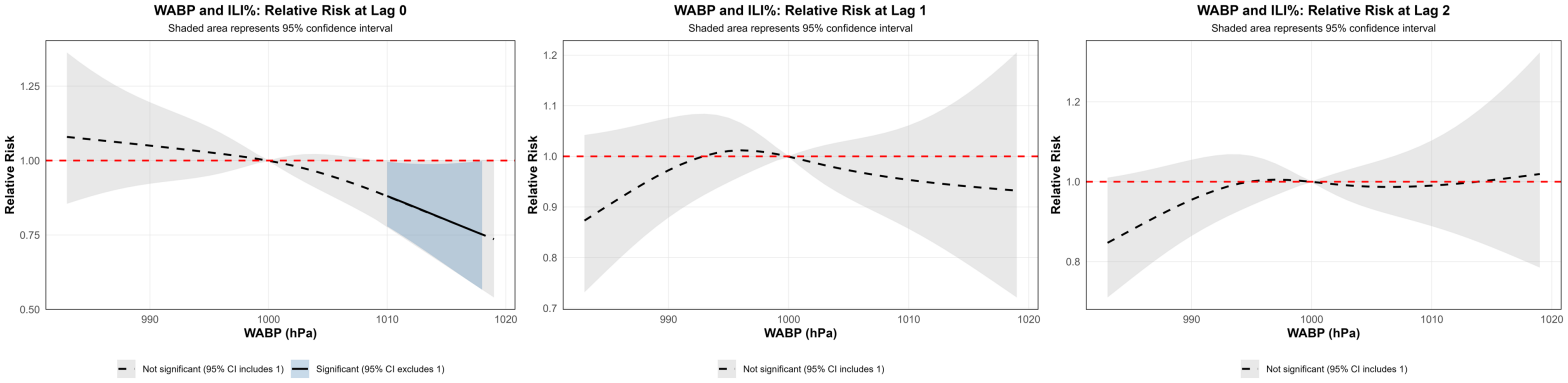
Non-linear and delayed associations of WABP with ILI% risk over a lag period of 0–3 weeks.

Using the median WABP (999.91 hPa) as the reference value and a maximum lag period of 3 weeks, we assessed the association between atmospheric pressure and ILI% risk in Quanzhou by means of 3D surface and contour plots (Figures 5a,b). The contour plot revealed a nonlinear exposure-response relationship, characterized by an elevated risk of ILI% at lower air pressure levels, whereas higher pressure exhibited a protective effect. Specifically, as shown in Figure 6, at shorter lag periods (0–0.5 weeks), air pressure below approximately 997 hPa was associated with an increase in ILI% risk, although this association was not statistically significant. For example, at 983 hPa and lag 0 week, the relative risk was 1.08 (95% CI, 0.85–1.36). In contrast, higher air pressure (≥1,010 hPa) during lag periods of 0–1.5 weeks showed statistically significant protective effects, with the relative risk (RR) values consistently below 1 and 95% confidence intervals excluding the null value (e.g., RR = 0.85, 95% CI: 0.73–0.99 at 1012 hPa and lag 0). These results indicate that the influence of air pressure on ILI% risk is dependent on both the pressure magnitude and the lag time, with moderate to high air pressure conferring a protective benefit.

#### 3.3.2 Effect of WAP on ILI%

**Figure 7:**
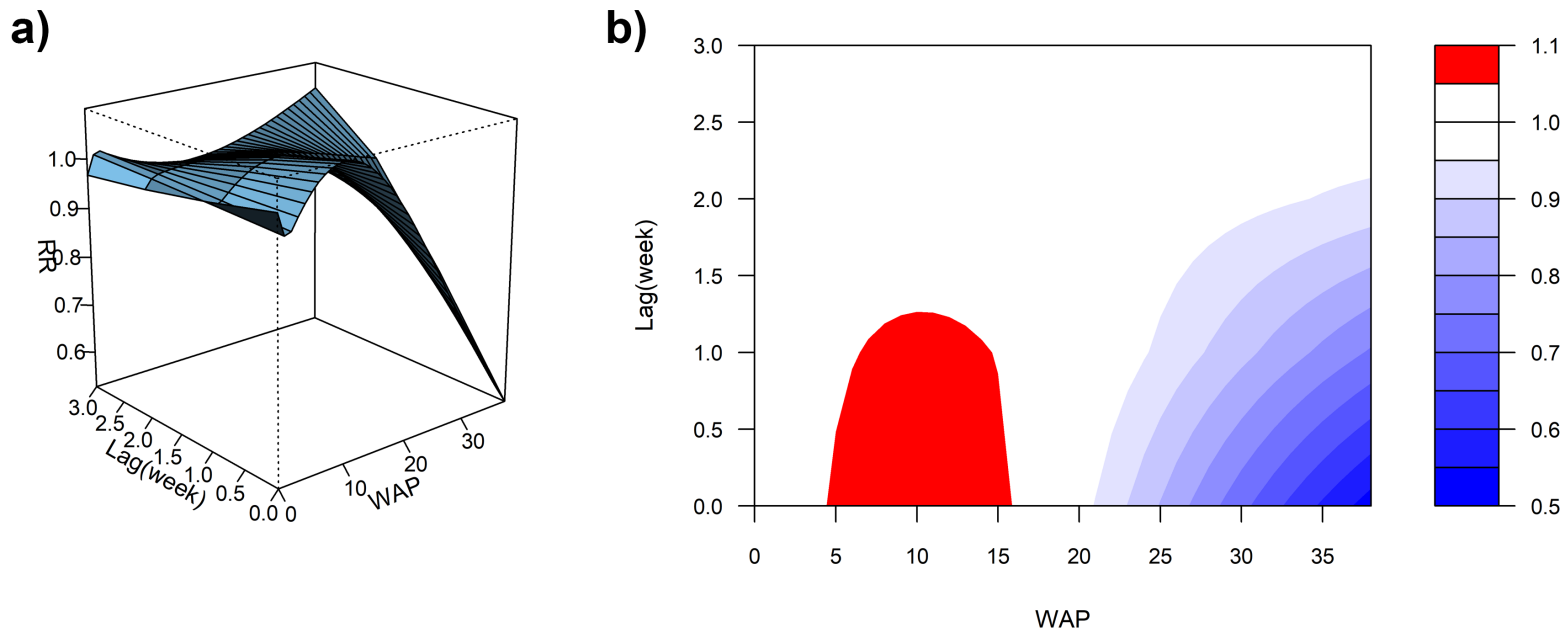
**(a)** 3D graph of WAP-lag-ILI% association; **(b)** contour graph of WAP-lag-ILI% association.

**Figure 8:**
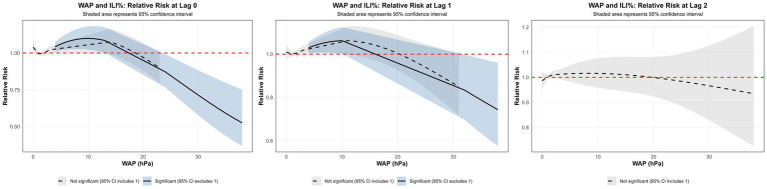
Non-linear and delayed associations of WAP with ILI% risk over a lag period of 0–3 weeks.

Using the median WAP (0.74 mm) as the reference and a maximum lag of 3 weeks, DLNM results revealed a significant non-linear association between precipitation and ILI% risk (Figures 7a,b, 8). When WAP ranged between 4 mm and 16 mm, RR consistently exceeded 1, indicating an elevated risk of ILI%. This effect was most pronounced at shorter lags (0 to 1.3 weeks). The highest risk occurred at a precipitation level of 10 mm at lag 0 week, with an RR of 1.10 (95% CI: 1.02–1.18). In contrast, precipitation levels exceeding 21 mm were associated with RR values below 1, suggesting a protective effect against ILI%.

#### 3.3.3 Influence of WAWS on ILI%

**Figure 9:**
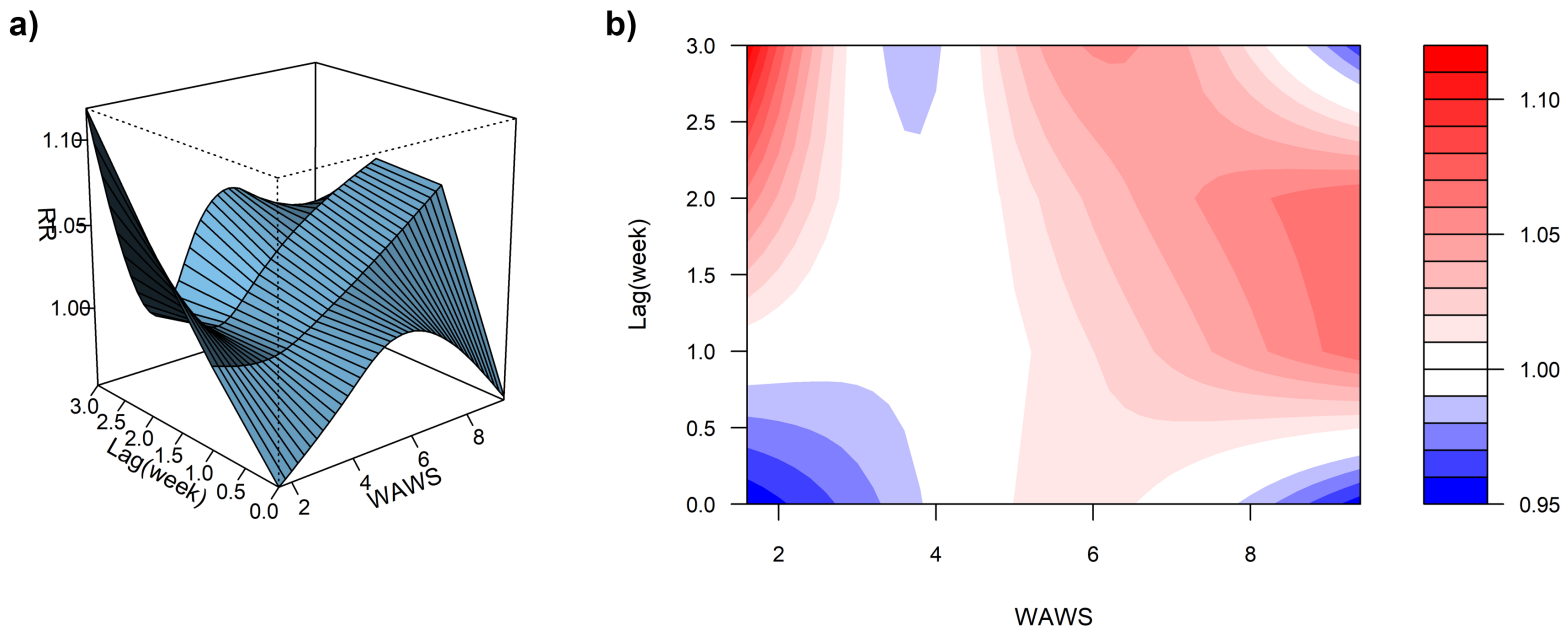
**(a)** 3D graph of WAWS-lag-ILI% association; **(b)** contour graph of WAWS-lag-ILI% association.

**Figure 10:**
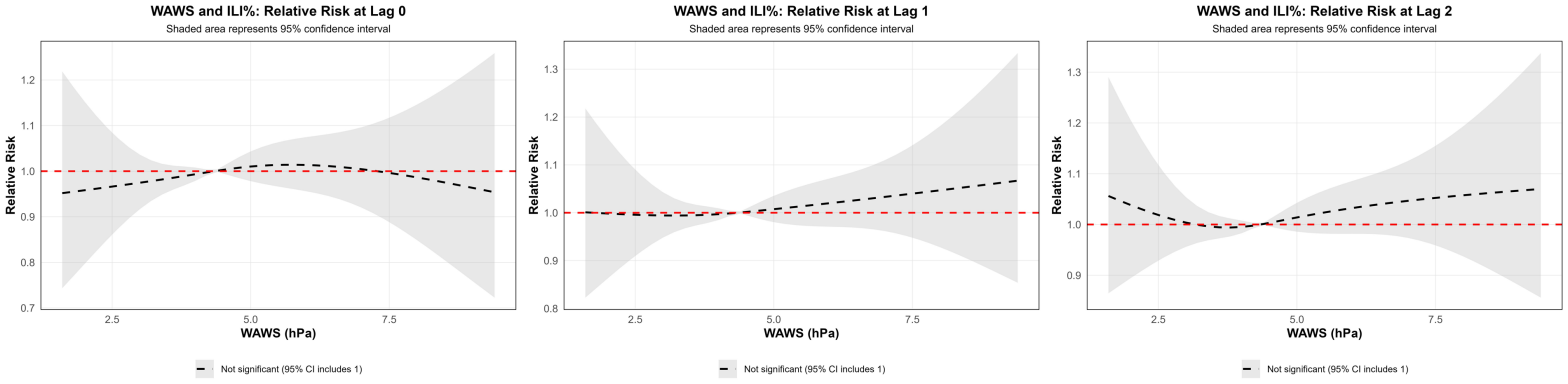
Non-linear and delayed associations of WAWS with ILI% risk over a lag period of 0–3 weeks.

Using the median WAWS of 4.34 m/s as the reference and a maximum lag period of 3 weeks, Figure 9a (3D surface plot) and Figure 9b (contour plot) suggest a nonlinear “N”-shaped exposure-response association between WAWS and the RR of ILI%. This “N-shaped” pattern indicates a non-monotonic relationship wherein ILI% risk initially increases with rising wind speed, subsequently decreases within a moderate range, and then increases again at higher wind speeds—forming a trajectory reminiscent of the letter “N” across exposure levels. Figure 10 further illustrates the lag patterns of WAWS over 0–3 weeks. At low wind speeds (< 3 m/s) and high wind speeds (> 4.5 m/s), the point estimates of RR were generally above 1, though most 95% confidence intervals included the null value (RR = 1). For instance, at a wind speed of 1.6 m/s and a lag of 3 weeks, the RR was 1.12 (95% CI, 0.87, 1.43). These results indicate suggestive trends rather than statistically significant increases in risk. A delayed pattern was observed at low wind speeds, with RR point estimates remaining above 1 across lag 1 to 3 weeks. At high wind speeds, the point estimates of RR rose more steeply with increasing wind speed, but again, confidence intervals frequently included (Figure 10). Thus, while the model indicates a potential nonlinear and delayed association, the overall evidence of association should be interpreted with caution due to the lack of statistical significance across most exposure levels and lags.

#### 3.3.4 Sensitivity analysis

To assess the robustness of our results, we conducted sensitivity analyses using different model specifications for the DLNM, including variations in the maximum lag period (2–4 weeks), degrees of freedom for exposure-response and lag-response functions (2–4 df), and the temporal control spline (5–8 df per year). As shown in Appendix 1, the estimated associations between meteorological factors and ILI% remained consistent across all sensitivity models. The maximum RRs for WAP ranged from 1.01 to 1.38, which overlapped with the main model estimate (RR = 1.10, 95% CI: 1.02–1.18). Similarly, results for WABP (RR range: 1.03–1.19) and WAWS (RR range: 1.10–1.46) showed comparable effect sizes and direction across specifications. The full results of the sensitivity analysis are presented in Appendix 1.

### 3.4 Forecasting results: univariate SARIMA vs. multivariate ARIMAX models

#### 3.4.1 Univariate SARIMA models

**Figure 11:**
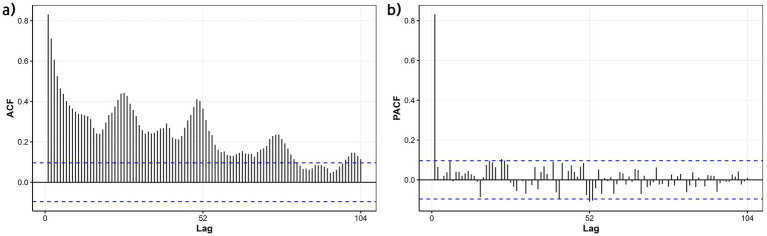
ACF and PACF plots of the differenced ILI% series in the training set (Quanzhou City, January 2016 to December 2023). **(a)** ACF; **(b)** PACF.

The time-series data from 2016 to 2023 were used as the training set to model influenza dynamics. Initial unit root testing (ADF) indicated stationarity in the ILI% series (*p* = 0.047); however, given the marginal significance near the critical threshold, a KPSS test was further conducted, confirming non-stationarity (*p* < 0.05). Visual inspection of autocorrelation function (ACF) plots revealed a slow decay pattern, characteristic of non-stationary data, necessitating first-order differencing. Post-differencing, the ILI% series achieved stationarity (*p* < 0.05), with the ARIMA model parameter *d* = 1. Strong seasonality was identified, marked by a 52-week cycle corresponding to annual influenza patterns. Autocorrelation (ACF) and partial autocorrelation (PACF) plots (Figure 11) indicated potential non-seasonal and seasonal autoregressive (*p*,*P*) and moving average (*q*,*Q*) orders of 1 or 2. Automated model selection via the auto.arima function in R, optimized by AIC and BIC, identified the seasonal ARIMA(1,1,1)(1,1,1)_52_ model as the optimal baseline configuration, with AIC = 747.86 and BIC = 767.33. The adequacy of this model was assessed by examining the residuals for autocorrelation using the Ljung-Box test. The results indicated no significant autocorrelation (*χ*^2^ = 23.94, *p* = 0.245), supporting that the residuals approximate white noise and that the model adequately captured the temporal structure of the data. The model demonstrated a good in-sample fit on the training data, with an RMSE of 0.599, a Mean Absolute Error (MAE) of 0.345, and a Mean Absolute Percentage Error (MAPE) of 18.67%. However, when evaluated on the out-of-sample test set (2024 data), the model’s predictive performance was lower, yielding an RMSE of 1.03, an MAE of 0.80, and a MAPE of 32.33%. These results indicate that while the model provided a reasonably accurate fit to the historical data, its generalizability to new data is limited, highlighting the potential need for incorporating exogenous variables to improve forecast accuracy.

#### 3.4.2 Multivariate ARIMAX modeling

CCF analysis was conducted on the training set (2016–2023) to identify optimal lag orders for WABP, WAP, and WAWS in relation to ILI%, with a maximum lag constraint of 3 weeks. Results indicated distinct lag patterns: WABP exhibited immediate effects (lag 0), WAP showed a delayed effect (lag 2 weeks), and WAWS demonstrated an immediate effect (lag 0).

The baseline seasonal ARIMA(1,1,1)(1,1,1)_52_ model, constructed without meteorological variables using the training set, achieved a training AIC of 747.86 and BIC of 767.33. When evaluated on the strictly held-out test set (2024 data), it yielded an RMSE of 1.03, an MAE of 0.80, and a MAPE of 32.33% (Table 2).

**Table 2:** Comparison of ARIMA (1,1,1)(1,1,1)_52_ models with and without meteorological factors.

Model	AIC	BIC	Test_RMSE	Test_MAE	Test_MAPE	RMSE improvement	MAE improvement
Baseline ARIMA (no predictors)	747.86	767.33	1.03	0.8	32.33	0%	0%
ARIMA + WABP(lag0)	737.6	760.93	1.06	0.83	33.36	−2.9%	−3.7%
ARIMA + WAP(lag2)	739.35	762.68	1.00	0.79	31.94	2.9%	1.3%
ARIMA + WAWS(lag0)	743.37	766.7	1.03	0.8	32.12	0.0%	0.0%
ARIMA + WABP(lag0) + WAP(lag2)	735.45	762.67	1.04	0.82	33.23	−1.0%	−2.5%
ARIMA + WABP(lag0) + WAWS(lag0)	737.97	765.2	1.07	0.83	33.41	−3.9%	−3.7%
ARIMA + WAP(lag2) + WAWS(lag0)	739.61	766.83	1.01	0.79	32.17	1.9%	1.3%
ARIMA + WABP(lag0) + WAP(lag2) + WAWS(lag0)	735.94	767.05	1.05	0.82	33.34	−1.9%	−2.5%

The best performing model is ARIMA + WAP(lag2), which improves RMSE by 2.9% and MAE by 1.3% compared to the baseline model.

These lag-optimized meteorological factors—identified exclusively from the training set—were subsequently incorporated as exogenous variables into the baseline model, either individually or in combination, generating seven candidate ARIMAX models. The forecasting performance of all candidate models is summarized in Table 2. Notably, the inclusion of exogenous variables did not uniformly enhance model performance. Four of the seven candidate models exhibited increased RMSE and MAE values compared to the baseline, highlighting that meteorological covariates may introduce noise unless judiciously selected and lag-optimized. For instance, the combined incorporation of WABP and WAWS at their respective optimal lags resulted in a 3.9% increase in RMSE relative to the baseline.

However, the model incorporating solely WAP at a 2-week lag (WAP(lag2)) emerged as an exception. When evaluated on the independent 2024 test set, this model achieved an RMSE of 1.00 and an MAE of 0.79, corresponding to a modest but consistent reduction in error metrics (2.9% in RMSE and 1.3% in MAE) compared to the baseline. It also attained the lowest MAPE (31.94%) among all configurations evaluated. The divergent performance across model configurations underscores the critical importance of variable-specific and lag-specific selection when integrating meteorological factors into forecasting models, and confirms that no information from the test set was used in model training or variable lag selection.

**Figure 12:**
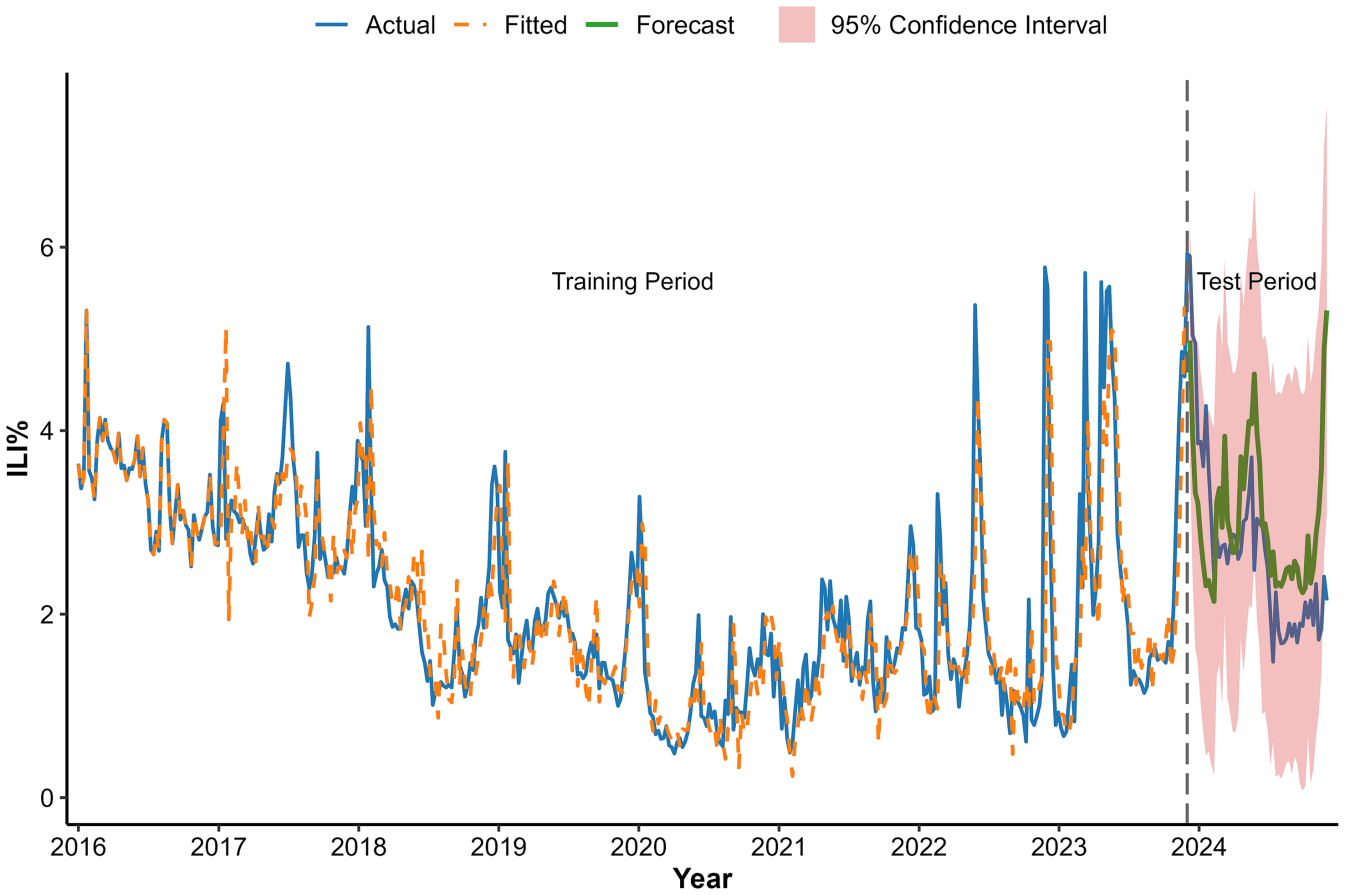
Forecast based on ARIMA(1,1,1)(1,1,1)_52_ + WAP(lag2) model fit.

The WAP(lag2) model demonstrated satisfactory forecast accuracy on the 2024 test data, with predicted ILI% values largely within the 95% confidence intervals and the forecast trend closely capturing the overall temporal dynamics of the observed data (Figure 12). The corresponding numerical values are tabulated in Appendix 2.

## 4 Discussion

The influenza-like illness percentage (ILI%) in Quanzhou City exhibited distinct temporal variations between 2016 and 2024, characterized by an initial significant decline from 2016 to 2020 followed by a rebound through 2024. This pattern likely reflects the substantial impact of non-pharmaceutical interventions (NPIs) implemented during the COVID-19 pandemic. Measures such as mask-wearing and social distancing not only suppressed SARS-CoV-2 transmission but also reduced influenza spread. Subsequent relaxation of NPIs, along with resumed social activities and changes in public health policies, probably facilitated the resurgence of influenza virus transmission (20). Age-specific incidence was highest among children under 5 years, followed by those aged 5–15 years, likely due to immunological naïveté, high contact rates in daycare and school settings, and group susceptibility. A clear seasonal pattern emerged, with a primary peak in winter (December–February) and a secondary peak in summer (May–July). Summer outbreaks may correlate with holiday cycles (e.g., May Day Golden Week and summer vacations), which intensify crowd gatherings and spatial proximity, enhancing transmission risks. Winter peaks, conversely, align with increased viral stability at low temperatures (21) and cold-induced suppression of respiratory immunity, compromising nasal mucosal barrier function (22). Additionally, indoor congregation during colder months further amplifies transmission opportunities.

Spearman correlation and distributed lag non-linear modeling (DLNM) identified significant associations between ILI% and several meteorological factors. Atmospheric pressure showed a U-shaped relationship with ILI risk: lower pressure (∼997 hPa) at shorter lags (0–0.5 weeks) increased risk, whereas higher pressure (≥ 1,010 hPa) was protective at slightly longer lags (0–1.5 weeks). These findings partially align with Zhu et al. (23), who reported increased risk under very low pressure (<980 hPa), suggesting a possible threshold effect. Conversely, the result that high pressure is protective contrasts with a previous domestic report (24) that indicated elevated risk. These discrepancies may be attributable to regional climatic differences or variations in modeling approaches. Moderate weekly precipitation (4–16 mm) was associated with elevated influenza incidence, which aligns with previous reports (25). This association can be explained by a combination of behavioral and physiological pathways. First, rainfall encourages indoor gathering, especially in crowded or inadequately ventilated settings, thereby facilitating close-contact transmission of respiratory viruses (26). The 2-week lag observed between precipitation events and the ILI% peak further suggests the contribution of physiological processes in addition to behavioral factors. Specifically, rainy conditions are often accompanied by diminished solar radiation, which can reduce cutaneous vitamin D synthesis. Vitamin D deficiency—common during winter—plays an important immunomodulatory role; its active form, 1,25(OH)₂D, enhances expression of antimicrobial peptides in respiratory epithelial cells and helps regulate inflammatory responses, thus strengthening lung defense against infection (27). The two-week interval corresponds to the period required for reduced sunlight to lower vitamin D levels, compromise immune function, and ultimately manifest as increased influenza activity at the population level. Notably, precipitation exceeding 21 mm was associated with a protective effect (RR < 1). Although this threshold is slightly below the national standard definition of heavy precipitation (≥25 mm), it likely reflects a comparable cleansing effect on airborne viral particles through rainfall-induced removal of suspended particulate matter (28, 29).

Similarly, weekly mean wind speed demonstrated a suggestive N-shaped exposure-lag-response association with ILI% risk. Model estimates indicated elevated RR values at both low (<3 m/s) and high (>4.5 m/s) wind speeds across certain lag periods, although these associations were not statistically significant, as most 95% confidence intervals included the null value. This pattern suggests a complex, non-monotonic relationship that merits cautious interpretation. A study by Qi L et al. (30) also reported that low wind speeds may increase influenza risk, potentially due to reduced air circulation leading to poor dispersion of viral particles and increased opportunities for transmission in poorly ventilated indoor settings where people tend to gather. On the other hand, high wind speeds were also associated with elevated RR in our model, a finding consistent with research by Zhou Yanli et al. (31), which proposed that strong winds may hinder the settling of virus-containing particles and facilitate their wider spatial dissemination, potentially expanding the population at risk.

In this study, weekly ILI% data from January 2016 to December 2023 were used as the training set, while data from January to December 2024 served as the test set. The optimal model was identified as the seasonal ARIMA(1,1,1)(1,1,1)_52_. While ARIMA models demonstrate utility in influenza prediction by leveraging historical incidence trends, their predictive efficacy exhibits significant regional variability. This limitation stems from the model’s inherent focus on time-series characteristics of incidence data, excluding external moderating variables such as environmental or socioeconomic factors. To address this, researchers have increasingly integrated meteorological parameters into influenza forecasting frameworks, with studies confirming the superior performance of ARIMAX models over traditional ARIMA approaches (32, 33).

We incorporated weekly mean atmospheric pressure, precipitation, and wind speed as exogenous variables into the baseline ARIMA model. Crucially, meteorological integration did not uniformly improve predictions; four of the seven ARIMAX models performed worse than the baseline, indicating that inappropriate inclusion of such variables can impair model accuracy. However, the model incorporating precipitation at a 2-week lag (WAP(lag2)) achieved modest but consistent improvements, suggesting that selectively and appropriately lagged meteorological factors may still offer predictive value.

These results emphasize that incorporating meteorological data requires rigorous lag structure selection and variable-specific validation. The overall mixed performance underscores that exogenous variables should be introduced cautiously to avoid introducing noise rather than signal.

Several limitations should be acknowledged. The analysis did not differentiate influenza subtypes, which may respond differently to meteorological conditions (34). The study period also included the COVID-19 pandemic, which may have distorted typical influenza-environment relationships due to non-pharmaceutical interventions (35). Additionally, socioeconomic confounders such as population mobility and vaccination coverage were not included. Future studies should thus pursue more comprehensive, multi-factor frameworks while validating meteorological associations across broader spatial and temporal contexts.

## 5 Conclusion

This study delineates the dynamic evolution of influenza-like illness (ILI%) in Quanzhou City from 2016 to 2024, revealing several major findings that enhance our understanding of influenza transmission patterns in a subtropical coastal city. Significant temporal trends were observed, characterized by a marked decrease in ILI% from 2016 to 2020 (APC = −22.693, *p* = 0.001), followed by a significant rebound after 2020 (APC = 21.555, *p* = 0.003). This biphasic pattern likely reflects the profound impact of COVID-19 containment measures and their subsequent relaxation. Clear epidemiological patterns were identified, with influenza transmission in Quanzhou exhibiting a consistent bimodal seasonal distribution featuring a primary peak in winter (December–February) and a secondary peak in summer (May–July). Higher incidence rates were consistently observed among children aged 0–15 years, particularly in nursery and school settings, highlighting this demographic as particularly vulnerable. From a modeling perspective, key meteorological factors—including atmospheric pressure, precipitation, and wind speed—showed significant nonlinear and lagged associations with ILI risk. Most notably, the incorporation of precipitation as an exogenous variable into an ARIMAX model significantly improved forecasting performance over conventional time-series models, underscoring the value of integrating environmental covariates into public health forecasting systems for respiratory infectious diseases.

## Data Availability

The raw data supporting the conclusions of this article will be made available by the authors, without undue reservation.
