# Supplementary material for: Improving influenza prediction in Quanzhou, China: an ARIMAX model integrated with meteorological drivers
Source: Front Public Health. 2025 Oct 22;13:1662775. doi: 10.3389/fpubh.2025.1662775 (PMC12586018; doi:10.3389/fpubh.2025.1662775)
Supplement: Supplementary file 1 [file Table_1.docx]

**Appendix 1: Sensitivity Analysis of DLNM Model Parameters: Maximum Cumulative Relative Risks (RR) for Associations between Meteorological Variables and ILI%**

| Variable | Max RR | CI_95_low | CI_95_high | Lag_weeks | Value | Model_Type | Parameters |
| --- | --- | --- | --- | --- | --- | --- | --- |
| **WABP** | **1.08** | **0.85** | **1.36** | **0** | **983** | **Main Model** | **lag=3, df_var=3, df_lag=3, time_df=7/year** |
| **WAP** | **1.1** | **1.02** | **1.18** | **0** | **10** | **Main Model** | **lag=3, df_var=3, df_lag=3, time_df=7/year** |
| **WAWS** | **1.12** | **0.87** | **1.43** | **3** | **1.6** | **Main Model** | **lag=3, df_var=3, df_lag=3, time_df=7/year** |
| WABP | 1.03 | 0.93 | 1.13 | 0 | 991 | Sensitivity | lag=2, df_var=2, df_lag=2, time_df=5/year |
| WAP | 1.15 | 0.87 | 1.52 | 2 | 38 | Sensitivity | lag=2, df_var=2, df_lag=2, time_df=5/year |
| WAWS | 1.21 | 0.96 | 1.51 | 2 | 9.4 | Sensitivity | lag=2, df_var=2, df_lag=2, time_df=5/year |
| WABP | 1.05 | 0.88 | 1.27 | 4 | 983 | Sensitivity | lag=4, df_var=2, df_lag=2, time_df=5/year |
| WAP | 1.18 | 0.92 | 1.52 | 4 | 38 | Sensitivity | lag=4, df_var=2, df_lag=2, time_df=5/year |
| WAWS | 1.25 | 1.05 | 1.5 | 4 | 9.4 | Sensitivity | lag=4, df_var=2, df_lag=2, time_df=5/year |
| WABP | 1.14 | 1.02 | 1.27 | 2 | 993 | Sensitivity | lag=2, df_var=4, df_lag=2, time_df=5/year |
| WAP | 1.34 | 0.97 | 1.84 | 2 | 38 | Sensitivity | lag=2, df_var=4, df_lag=2, time_df=5/year |
| WAWS | 1.1 | 0.96 | 1.27 | 2 | 8 | Sensitivity | lag=2, df_var=4, df_lag=2, time_df=5/year |
| WABP | 1.11 | 1.01 | 1.22 | 0 | 993 | Sensitivity | lag=4, df_var=4, df_lag=2, time_df=5/year |
| WAP | 1.38 | 1.04 | 1.84 | 4 | 38 | Sensitivity | lag=4, df_var=4, df_lag=2, time_df=5/year |
| WAWS | 1.19 | 0.94 | 1.52 | 4 | 9.4 | Sensitivity | lag=4, df_var=4, df_lag=2, time_df=5/year |
| WABP | 1.11 | 0.86 | 1.45 | 0 | 983 | Sensitivity | lag=4, df_var=2, df_lag=4, time_df=5/year |
| WAP | 1.16 | 0.87 | 1.54 | 3 | 38 | Sensitivity | lag=4, df_var=2, df_lag=4, time_df=5/year |
| WAWS | 1.22 | 1.01 | 1.47 | 2 | 9.4 | Sensitivity | lag=4, df_var=2, df_lag=4, time_df=5/year |
| WABP | 1.15 | 0.97 | 1.37 | 0 | 990 | Sensitivity | lag=4, df_var=4, df_lag=4, time_df=5/year |
| WAP | 1.36 | 0.98 | 1.9 | 3 | 38 | Sensitivity | lag=4, df_var=4, df_lag=4, time_df=5/year |
| WAWS | 1.23 | 0.91 | 1.67 | 4 | 9.4 | Sensitivity | lag=4, df_var=4, df_lag=4, time_df=5/year |
| WABP | 1.15 | 0.96 | 1.37 | 0 | 983 | Sensitivity | lag=2, df_var=2, df_lag=2, time_df=8/year |
| WAP | 1.06 | 0.84 | 1.34 | 2 | 38 | Sensitivity | lag=2, df_var=2, df_lag=2, time_df=8/year |
| WAWS | 1.17 | 0.95 | 1.42 | 2 | 9.4 | Sensitivity | lag=2, df_var=2, df_lag=2, time_df=8/year |
| WABP | 1.07 | 0.91 | 1.26 | 0 | 983 | Sensitivity | lag=4, df_var=2, df_lag=2, time_df=8/year |
| WAP | 1.05 | 0.83 | 1.33 | 4 | 38 | Sensitivity | lag=4, df_var=2, df_lag=2, time_df=8/year |
| WAWS | 1.41 | 1.18 | 1.7 | 4 | 9.4 | Sensitivity | lag=4, df_var=2, df_lag=2, time_df=8/year |
| WABP | 1.15 | 0.96 | 1.39 | 0 | 983 | Sensitivity | lag=2, df_var=4, df_lag=2, time_df=8/year |
| WAP | 1.13 | 0.86 | 1.49 | 2 | 38 | Sensitivity | lag=2, df_var=4, df_lag=2, time_df=8/year |
| WAWS | 1.18 | 0.92 | 1.52 | 2 | 9.4 | Sensitivity | lag=2, df_var=4, df_lag=2, time_df=8/year |
| WABP | 1.06 | 0.94 | 1.19 | 0 | 988 | Sensitivity | lag=4, df_var=4, df_lag=2, time_df=8/year |
| WAP | 1.08 | 0.82 | 1.44 | 4 | 38 | Sensitivity | lag=4, df_var=4, df_lag=2, time_df=8/year |
| WAWS | 1.42 | 1.11 | 1.81 | 4 | 9.4 | Sensitivity | lag=4, df_var=4, df_lag=2, time_df=8/year |
| WABP | 1.19 | 0.96 | 1.47 | 0 | 983 | Sensitivity | lag=4, df_var=2, df_lag=4, time_df=8/year |
| WAP | 1.01 | 1 | 1.02 | 3 | 0 | Sensitivity | lag=4, df_var=2, df_lag=4, time_df=8/year |
| WAWS | 1.34 | 1.08 | 1.66 | 4 | 9.4 | Sensitivity | lag=4, df_var=2, df_lag=4, time_df=8/year |
| WABP | 1.11 | 0.88 | 1.41 | 0 | 983 | Sensitivity | lag=4, df_var=4, df_lag=4, time_df=8/year |
| WAP | 1.02 | 0.75 | 1.39 | 3 | 38 | Sensitivity | lag=4, df_var=4, df_lag=4, time_df=8/year |
| WAWS | 1.46 | 1.12 | 1.9 | 1 | 9.4 | Sensitivity | lag=4, df_var=4, df_lag=4, time_df=8/year |

**Appendix 2: Comparison of Predicted and Observed ILI% Values for 2024 - Optimal ARIMAX Model with Precipitation (WAP) at 2-week Lag**

| Year | Week | Observed_ILI | Predicted_ILI | CI_Lower_95 | CI_Upper_95 |
| --- | --- | --- | --- | --- | --- |
| 2024 | 1 | 5.9 | 4.98 | 3.73 | 6.23 |
| 2024 | 2 | 5.04 | 3.97 | 2.43 | 5.52 |
| 2024 | 3 | 4.95 | 3.33 | 1.65 | 5.01 |
| 2024 | 4 | 3.86 | 3.21 | 1.45 | 4.96 |
| 2024 | 5 | 3.88 | 2.83 | 1.03 | 4.63 |
| 2024 | 6 | 3.61 | 2.54 | 0.72 | 4.37 |
| 2024 | 7 | 4.27 | 2.31 | 0.46 | 4.15 |
| 2024 | 8 | 3.57 | 2.37 | 0.5 | 4.23 |
| 2024 | 9 | 3.03 | 2.24 | 0.36 | 4.12 |
| 2024 | 10 | 2.41 | 2.14 | 0.25 | 4.03 |
| 2024 | 11 | 2.83 | 3.23 | 1.33 | 5.13 |
| 2024 | 12 | 2.62 | 3.37 | 1.46 | 5.28 |
| 2024 | 13 | 2.74 | 2.94 | 1.03 | 4.86 |
| 2024 | 14 | 2.76 | 3.94 | 2.01 | 5.87 |
| 2024 | 15 | 2.55 | 3.03 | 1.09 | 4.96 |
| 2024 | 16 | 2.75 | 2.87 | 0.92 | 4.81 |
| 2024 | 17 | 2.87 | 2.67 | 0.72 | 4.62 |
| 2024 | 18 | 2.85 | 2.68 | 0.72 | 4.64 |
| 2024 | 19 | 2.6 | 2.84 | 0.88 | 4.81 |
| 2024 | 20 | 2.68 | 3.72 | 1.74 | 5.69 |
| 2024 | 21 | 3.01 | 3.36 | 1.38 | 5.35 |
| 2024 | 22 | 3.04 | 3.75 | 1.76 | 5.74 |
| 2024 | 23 | 3.31 | 4.1 | 2.1 | 6.1 |
| 2024 | 24 | 3.71 | 4.07 | 2.07 | 6.08 |
| 2024 | 25 | 2.48 | 4.62 | 2.6 | 6.63 |
| 2024 | 26 | 3.04 | 3.98 | 1.95 | 6 |
| 2024 | 27 | 2.98 | 3.65 | 1.61 | 5.68 |
| 2024 | 28 | 2.98 | 2.95 | 0.91 | 4.99 |
| 2024 | 29 | 2.7 | 2.99 | 0.94 | 5.03 |
| 2024 | 30 | 2.49 | 2.8 | 0.74 | 4.85 |
| 2024 | 31 | 2 | 2.42 | 0.36 | 4.48 |
| 2024 | 32 | 1.48 | 2.68 | 0.61 | 4.75 |
| 2024 | 33 | 2.24 | 2.3 | 0.23 | 4.38 |
| 2024 | 34 | 1.82 | 2.34 | 0.26 | 4.43 |
| 2024 | 35 | 1.68 | 2.3 | 0.21 | 4.4 |
| 2024 | 36 | 1.69 | 2.38 | 0.28 | 4.48 |
| 2024 | 37 | 1.74 | 2.49 | 0.38 | 4.6 |
| 2024 | 38 | 1.9 | 2.51 | 0.4 | 4.63 |
| 2024 | 39 | 1.76 | 2.39 | 0.26 | 4.51 |
| 2024 | 40 | 1.89 | 2.58 | 0.45 | 4.71 |
| 2024 | 41 | 1.69 | 2.51 | 0.37 | 4.65 |
| 2024 | 42 | 1.9 | 2.3 | 0.15 | 4.44 |
| 2024 | 43 | 1.87 | 2.23 | 0.08 | 4.39 |
| 2024 | 44 | 2.13 | 2.29 | 0.13 | 4.45 |
| 2024 | 45 | 1.96 | 2.85 | 0.68 | 5.02 |
| 2024 | 46 | 2.15 | 2.34 | 0.16 | 4.51 |
| 2024 | 47 | 1.88 | 2.5 | 0.32 | 4.69 |
| 2024 | 48 | 2.33 | 2.87 | 0.68 | 5.06 |
| 2024 | 49 | 1.72 | 3.12 | 0.92 | 5.31 |
| 2024 | 50 | 1.84 | 3.59 | 1.38 | 5.79 |
| 2024 | 51 | 2.41 | 4.89 | 2.67 | 7.1 |
| 2024 | 52 | 2.15 | 5.31 | 3.09 | 7.53 |

This table presents the weekly comparison between predicted ILI% values from the optimal ARIMAX model (ARIMA(1,1,1)(1,1,1)52 with weekly average precipitation at 2-week lag) and the actual observed ILI% values for the test period (2024). The model demonstrates satisfactory forecasting performance with 92.3 % of observations falling within the 95% prediction interval.
